# Ablation Characteristics Research in Solid Rocket Motor’s Combustion Chamber Produced by 3D Printing

**DOI:** 10.3390/ma16083021

**Published:** 2023-04-11

**Authors:** Weihua Hui, Yun Hu, Yang Liu, Qiang Cai, Weijie Zhao

**Affiliations:** 1 Science and Technology on Combustion Internal Flow and Thermal-Structure Laboratory, Northwestern Polytechnical University, Xi’an 710072, China; 2 China Academy of Launch Vehicle Technology, Beijing 100076, China

**Keywords:** PA12/GBs, SRM, ablation characteristics, 3D printing

## Abstract

A polyamide 12(PA12) reinforced with glass beads (GBs) solid rocket motor (SRM) produced by 3D Printing is proposed in the paper. The ablation research of the combustion chamber is studied by simulating the motor’s operating environment through ablation experiments. The results show that the maximum ablation rate for the motor was 0.22 mm/s, which occurred at the location where the combustion chamber meets the baffle. The closer to the nozzle, the greater its ablation rate. Through the microscopic appearance analysis of the composite material from the inner wall surface to the outer wall surface in several directions before and after the ablation experiments, it was found that the GBs with weak or no interfacial adhesion to PA12 may make the mechanical properties of the material degrade. The ablated motor had a large number of holes and some deposits on the inner wall surface. Also by evaluating the surface chemistry of the material, it was found that the composite material underwent thermal decomposition. Moreover, it underwent a complex chemical reaction with the propellant.

## 1. Introduction

Over the past decade, interest and investment in space exploration have increased dramatically which has led to greater demand for satellites. As a propulsion system of satellites, SRMs have the advantages of simple structure, convenient maintenance, low cost, and high reliability, and they are also widely used in other aerospace fields [1,2]. At present, reducing the cost of launching satellites into space has become a hot issue for many companies. For the SRMs, reducing the quality of the SRMs’ shell can not only reduce the cost but also increase the specific impulse when other conditions remain unchanged.

Three-D printing technology doesn’t need to consider the geometric complexity of the model and it can easily create a free-form design through topology optimization [3,4]. Selective laser sintering (SLS) is one of the most widely used 3D printing technologies [5]. SLS is a rapid prototyping technology that creates 3D objects by sintering powder materials layer-by-layer using infrared laser beams [6,7,8]. The advantages of SLS include a wide selection of materials, high precision, complex components or dies, no support structure, and a high utilization rate of materials [9]. Polyamide is currently the most widely used polymer for laser sintering, especially PA12 which is the most commercial material. PA12 is very suitable for SLS owing to its low processing temperatures, low laser power requirement and high accuracy [10,11]. Compared with other thermoplastics, PA12 has strong toughness, fatigue resistance, good tribological properties, high chemical resistance, and so on [12,13]. Now, PA12 reinforced with glass beads parts printed by SLS has attracted much attention. The addition of glass beads enables the material to exhibit enhanced stiffness because they improve the bearing capacity within the elastic deformation range [14].

Ablation of SRMs is of great concern during operation. The chemical and mechanical erosion for ablation and ablation of composite materials is a complex process that involves both external and internal factors. This study focused on the effects of external factors such as combustion chamber [15], combustion temperature [16], nozzle geometry [17], non-homogeneous chemical reactions on the nozzle surface [18], the effect of particles on the nozzle throat [19], etc. As an important component of an SRM, the combustion chamber provides a place for the storage and combustion of the propellant. The propellant produces gas with temperatures up to 2000–3900 K and pressures up to 3–20 MPa. Therefore, it is very vital to study the ablation characteristics of the combustion chamber of the 3D-printed SRM.

The ablative material studied by Liwu Wang et al. [20] is a carbon–carbon composite woven with radial carbon rods. The preform is extremely complex. After the formation of the preform, the C/C composite is manufactured by high-temperature pretreatment, chemical vapor deposition, pitch impregnation and high-pressure carbonization, and graphitization, in turn. The data shows that the average erosion rate of C/C composites with high chemical vapor deposition (CVD) carbon content is 0.35 mm/s. Jiang Li et al. [21] investigated the ablation and erosion characteristics of ethylene propylene diene monomer (EPDM) composites under realistic solid rocket motor operating conditions using an ablation motor and an overload simulation erosion motor. The EPDM, phenolic resin, aramidfiber, silica, and other fillers were prepared using an XK-160 mixing roll mill according to the specific formulation proportions. Zinc borate, sulfur, and the vulcanization accelerator were then added, and the mixture was milled several times and then left to stand for 24 h. After mixing the rubber for 24 h, special tools were used to cut, mold, and vulcanize the rubber. This is the manufacturing process of this material. Silicafillers and aramidfibers have important effects on the ablation resistance of EPDM composites. The ablation resistance properties of non-silica and non-fiber formulations are obviously poor and worsen under erosion conditions with dense particle jets. From the analysis of the morphology and structure of the composite char layers, the combined use of silica and aramidfibers can make the char layer form a uniform network-like structure with a compact surface and a loose interior, improving both the heat-shielding and erosion-resistance performances of the char layer. Allcorn et al. investigated the potential of thermoplastic polyurethane elastomer nanocomposites as an alternative elastomeric heat-shielding material in ablation applications [22]. Three different nanofillers were tested, and small-scale ablation testing was performed using an oxyacetylene test bed. The results showed that the TPUN ablatives performed well relative to the baseline EHSM. Vaia et al. investigated the ablative performance of nylon/nanoclay nanocomposites [23]. This research showed that a relatively tough, inorganic char forms during the ablation of these nylon-clay nanocomposites resulting in at least an order-of-magnitude decrease in mass loss (erosion) rate relative to the neat polymer. This occurs for a small amount of 2 wt% (~0.8 vol%) exfoliated mica-type layered silicate. The nanoscopic distribution of silicate layers leads to a uniform char layer that enhances the ablative performance.

In summary, relevant research in the field of solid rocket motors has focused on the ablation analysis of different ablation-resistant materials. Although the results of these researches are informative, the understanding of ablation characteristics of solid rocket motor’s combustion chamber produced by 3D printing is limited. In this paper, we first designed a simple solid rocket motor and analyzed the variation of the combustion chamber pressure and the temperature of the outer combustion wall surface under ignition conditions by means of a pressure sensor and an infrared thermal imaging camera. In order to study the ablation characteristics of the combustion chamber, thermogravimetric (TG) analysis is performed on the material. Finally, the microscopic morphology of several surfaces of the SRM before and after ablation and the elemental content of different locations of the motor are explored to give a basis for the ablation mechanism of PA12/GB composites of solid rocket motors under high temperature and high-pressure conditions.

## 2. Materials and Methods

### 2.1. Materials and Models

**Figure 1:**
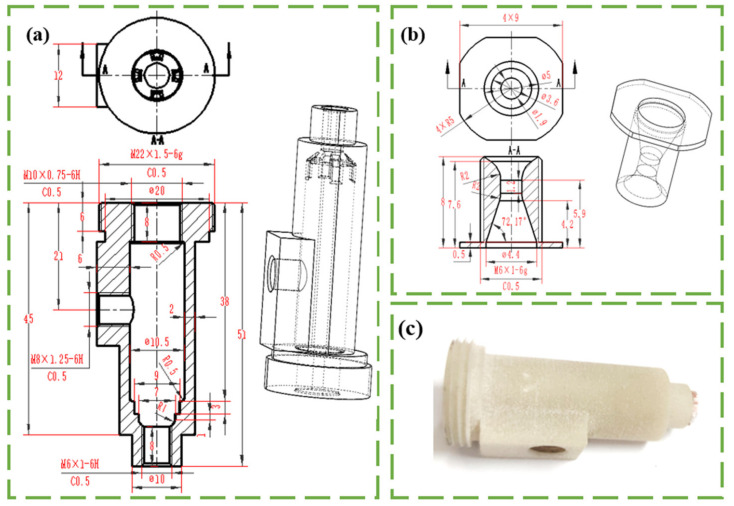
Schematic diagram of the motor: (**a**): shell produced by 3D printing, (**b**): nozzle manufactured by machining, (**c**): object picture.

To compensate for throat ablation and pay more attention to combustion chamber ablation, the nozzle is machined from metal materials and the shell is 3D-printed. The material of the nozzle is stainless-steel-304-annealed. The material of the shell is PA12/GBs. The PA12/GBs composite powder, supplied by the EOS Group, has the commercial named PA3200 GF for the reinforced PA12 with 40%wt of glass beads with an average size of 60 µm. The SLS samples for the study were manufactured by Creators Alliance Network Technology Co. (Dongguan, China). The relevant parameters are shown in Table 1. Figure 1 shows the schematic diagram of the motor used in the experiment. The nozzle is threaded into the rest of the motor, and the chamber is then filled with the solid propellant. The 22 mm radius thread is for assembly to the testbed, the 10 mm radius thread is for assembly to the thrust sensor, the 8 mm radius thread is for assembly to the pressure sensor, and the 6 mm radius thread is for assembly to the metal nozzle.

**Figure 2:**
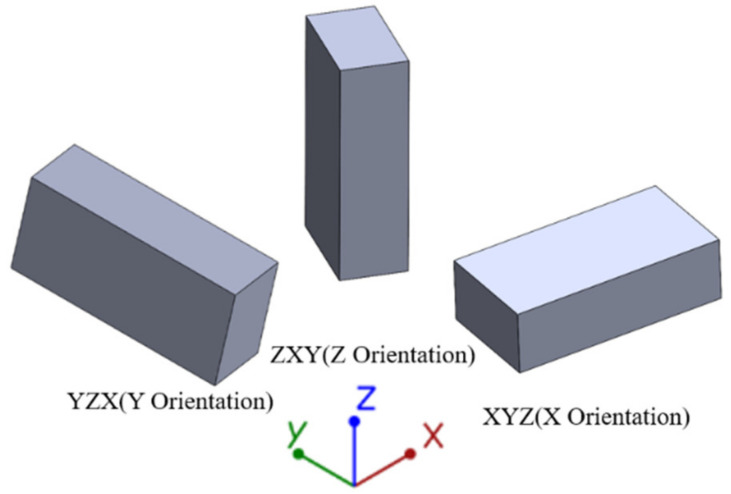
Schematic of type V dog bones in three build orientation axes.

The printed motor was printed using Performics’s laser powder bed fusion apparatus. The equipment was equipped with a CO_2_ laser unit. The apparatus was equipped with Buildstar control software that directly reads the STL files of the model. The characteristic resolution of SLS was mainly determined by laser beam diameter, laser power, powder deposition thickness, preheating temperature, and scan speed. Table 2 provides the finally determined process parameters for the printed device. To remove excess powders on the surface of the PA12/GBs parts, an abrasive blasting treatment was performed. Looking at three build orientations XYZ, YZX, and ZYX, simplified to X, Y, and Z from here in Figure 2, the shell was printed in the Z orientation.

### 2.2. Ablation Experiment

**Figure 3:**
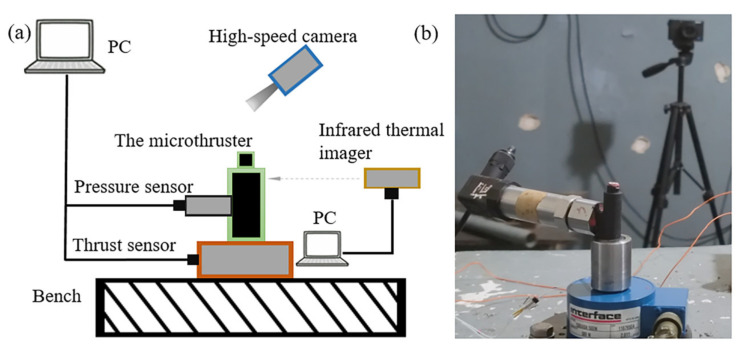
Ablation simulation experimental setup: schematic diagram on the left (**a**) and physical drawing on the right (**b**).

Double cobalt 2 (SG-2) propellant was chosen as the propellant, and its main formulations are shown in Table 3. During the ablation process, the maximum temperature of the inner wall surface of the motor is up to 2500 K. The ablation simulation experimental setup is shown in Figure 3. A data acquisition module including pressure and thrust sensors and a signal processor is used to obtain the pressure and thrust, a high-speed camera records the ignition phenomenon, and an infrared thermal imaging camera records the temperature of the motor’s outer wall surface. Note that in the paper, the ablation experiment is also referred to as the ignition experiment.

### 2.3. Characterisation

High-density materials and pore network models of the printed parts were collected using a high-resolution micro-computed tomography (micro-CT) system (Aoyun Electronic Technology Co., Shenzhen, China, AX2000).VG software was used to visually analyze the reconstructed 3D CT data of the printed components. The morphology was observed by field emission scanning electron microscopy (FESEM, FEI-NOVANANO 230, Corvallis, OR, USA) with an acceleration voltage of 5 kV. Prior to the optical examination, a thin conductive gold coating was applied by sputtering. And the energy dispersive spectrometer (EDS, X—MAX50, Oxford, UK) of the SEM is used to analyze the elemental composition of the motors. A dynamic thermogravimetric (TG) analysis (Nike Instrument Manufacturing Co., Selb, Germany, 209F3) of the PA12/GBs parts was performed at a heating rate of 10 °C/min from 20 to 600 °C in a nitrogen atmosphere, and the mass of the samples was ∼10 mg. X-ray photoelectron spectroscopy (XPS, Kratos Axis Supra, Shimadzu, Japan) was used to assess surface chemistry in the material. Spectra were curve resolved with CasaXPS 2.3.25PR1.0 software.

## 3. Results and Discussion

### 3.1. Ablation Analysis

**Figure 4:**
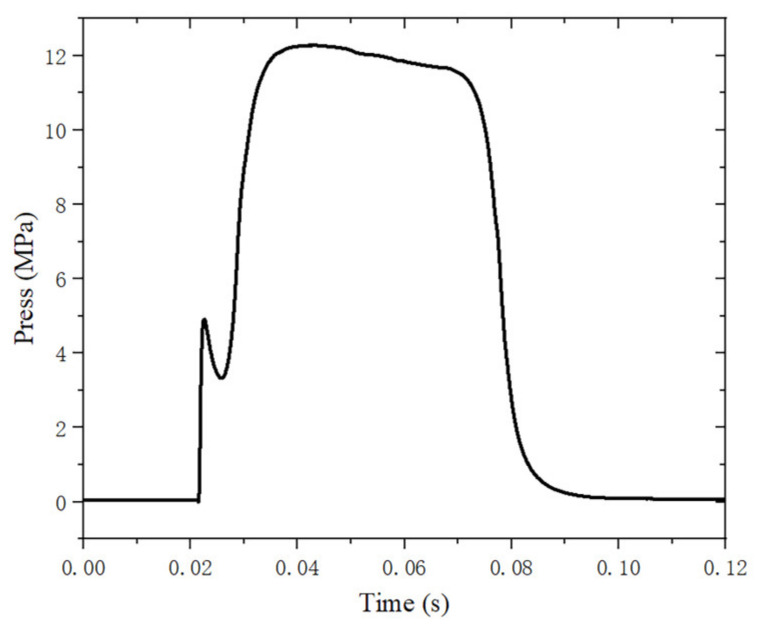
Variation of pressure with time.

Figure 4 shows the pressure-time curve of the motor. The starting point of ignition is when the pressure rises continuously to 0.3 MPa after the motor ignition, and the end point of ignition is when the pressure drops continuously to 0.3 MPa. It can be seen that the average pressure is about 12 MPa and the total operating time of ignition is 66 ms. The curve has an equilibrium section where the pressure slowly decreases, and then the pressure slowly decreases until the end of the ignition process, which shows that the motor successfully performs ground ignition under high temperature and pressure conditions.

**Figure 5:**
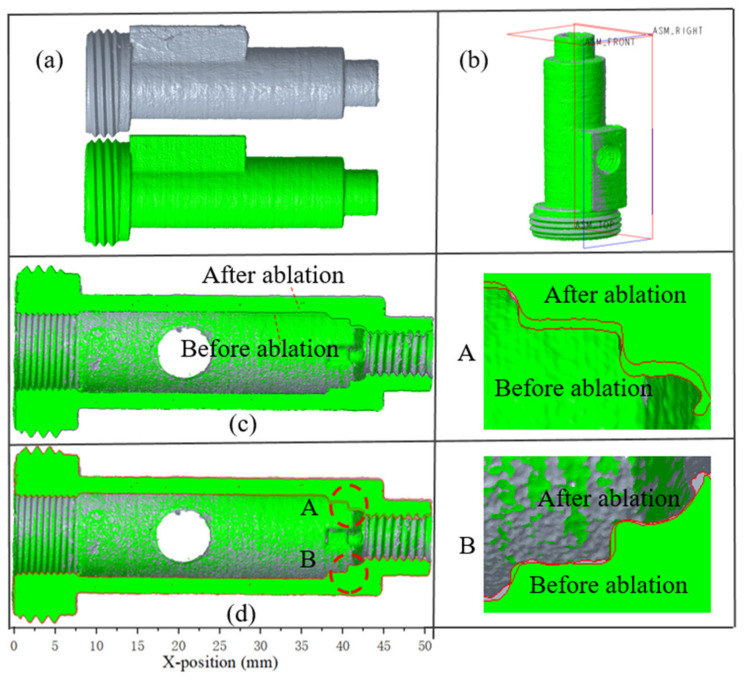
The 3D CT models of the engines. (**a**): the motors before and after ablation; (**b**): Overlay picture; (**c**,**d**): cross-sectional view, (**c**) is unmarked contour line and (**d**) is marked contour line; (A,B): detail diagram at the baffle.

W.-H. Hui et al. overlapped the three-dimensional reconstruction of the C/C composite nozzles before and after ablation and analyzed the nozzle ablation in detail [24]. To investigate the internal ablation, this method was used to analyze the ablation of the combustion chamber of the engine before and after the ablation. The 3D CT models of the engines before and after ablation were visualized and analyzed using VG software. By placing the two engines in one interface of the VG software, the two engines can be overlapped using the VG software’s automatic center of gravity recognition function. To distinguish the engines, the color of the pre-ablation engine is rendered as gray and the post-ablation engine is rendered as green (Figure 5a). The three datum planes of the engine are shown in Figure 5b. The front view datum plane is selected for offset, and the engine section view can be obtained, as shown in Figure 5c. In the measurement process of the linear ablation rate, the axis of the engine was selected as the baseline for the measurement. The difference between the diameter of each location on the inner wall surface of the motors from the front to the baffle was determined, using the measurement function in the VG software, and then divided by the operating time to represent the linear ablation rate. Among them, the measurement error is 0.015 mm.

The black line in Figure 5c is the contour line of the motor after ablation, and Figure 5d shows the contour line of the motors before and after ablation with red lines for a clear representation of the motor after ablation. The detail magnification of sections A and B shows that the ablation at the baffle is extremely obvious. The main reason is that, on the one hand, the molten particles hit the wall of the baffle under the impetus of high-speed airflow, which leads to mechanical damage and peeling of the composite material, and the scouring effect of the particles also enhances the heat transfer of the ablated material. On the other hand, the temperature of the material surface rises rapidly at the moment the solid particles hit the baffle, which also makes the thermochemical ablation of the material easier due to the rapid combustion of propellant, which generates a high-speed high-temperature airflow, and then this airflow scours and ablates the baffle.

**Figure 6:**
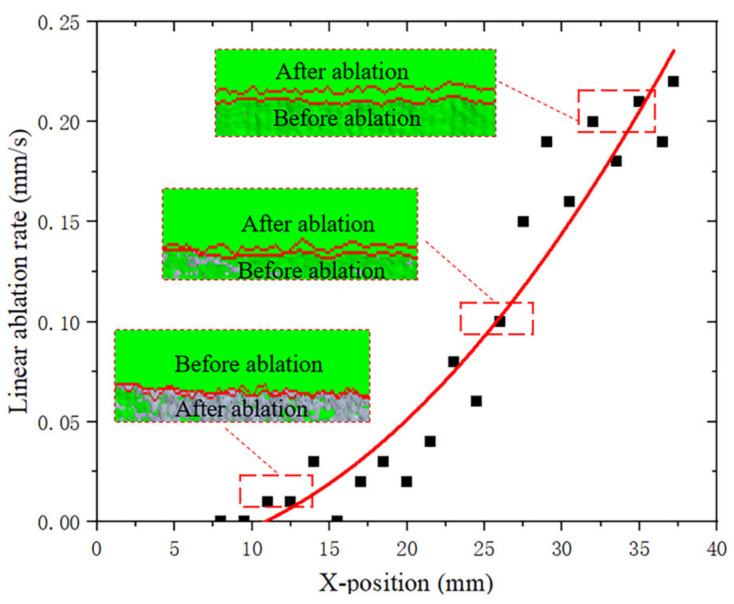
Ablation rate of the combustion chamber.

Neglecting the expansion characteristics of the material, the linear ablation rate of the combustion chamber is shown in Figure 6. As the distance from the nozzle shortens, the ablation rate at the combustion chamber increases. This phenomenon can be attributed to the part near the nozzle, where the passage area becomes smaller and the airflow velocity becomes larger, making the combustion velocity increase. At the same time, because the propellant combustion surface is no longer a complete and smooth solid boundary, but microscopically uneven, therefore, the mechanical scouring effect of the airflow will also accelerate the surface flaking, affecting the increase in combustion speed. The maximum ablation rate was 0.22 mm/s, which occurred at the position of the interface between the combustion chamber and the baffle.

### 3.2. Analysis of Microscopic Appearance

**Figure 7:**
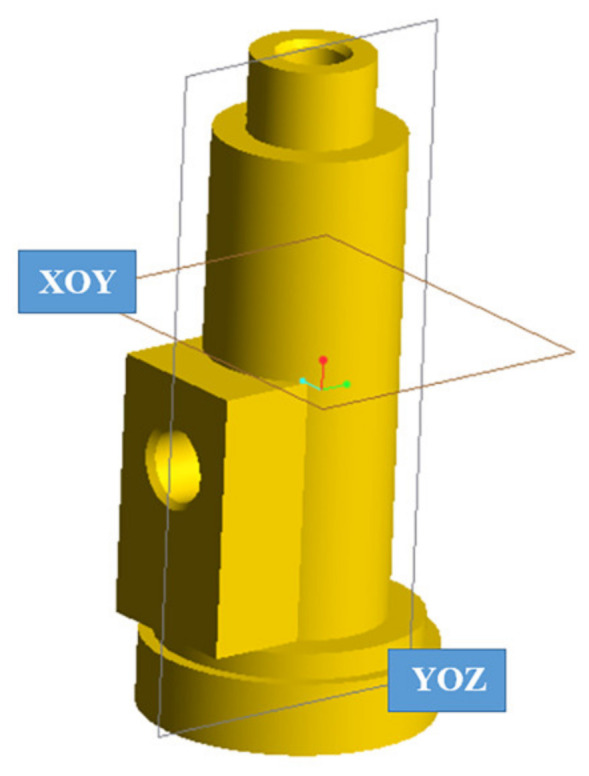
Position of the motor cutting plane.

To better investigate and analyze the motor ablation, the motor was cut along the XOY plane to observe the microscopic morphology from the inner wall surface to the outer wall surface in the transverse plane before and after ablation; the motor was cut along the YOZ plane to observe the microscopic morphology from the inner wall surface to the outer wall surface in the longitudinal plane before and after ablation. The cutting plane position is shown in Figure 7.

**Figure 8:**
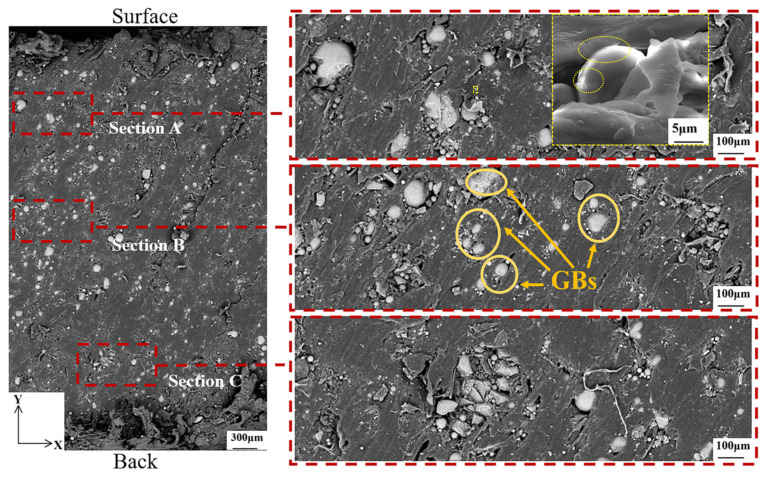
Microscopic morphology of the motor before ablation in the XOY direction. The larger yellow dashed rectangle is an enlarged view of the smaller yellow dashed rectangle.

To better compare the microscopic morphology of the motor from the inner wall surface to the outer wall surface before and after the ablation, three areas were taken for each plane for analysis, as shown in Figure 8, where “back” denotes the inner surface and “surface” denotes the outer surface. It can be seen that the glass balls used as fillers are not the same size. It is worth noting that the GBs shown by the yellow arrow are scattered or concentrated by PA12. Clusters of glass beads with poor (or no) adhesion between them may act as defects, suppressing toughness [14]. The magnified image in section A of the figure shows the presence of traces of molten PA12 on the GBs and the strong interfacial adhesion observed between PA12 and GBs. Because the bonding action of PA12 and GBs consumes high energy and the bonding force presents inside the molten PA12, the stiffness of PA12/GBs composites is improved [14].

**Figure 9:**
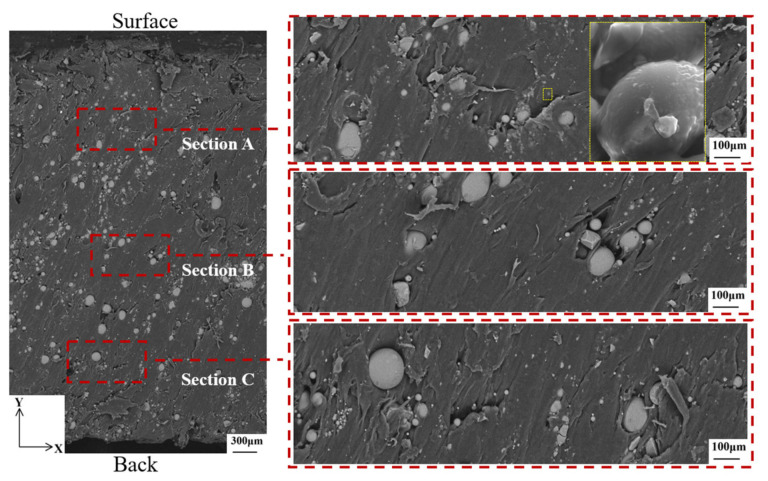
Microscopic morphology of the motor after ablation in the XOY direction. The larger yellow dashed rectangle is an enlarged view of the smaller yellow dashed rectangle.

After ablation, the morphology is not consistent with that before ablation because 3D printing does not produce two identical pieces. Although the basic dimensions are controlled to be the same and the position taken for cutting the sample is the same, the flow of the melting material during the printing process is not done consistently. The enlarged image in section A of Figure 9 showed that the morphology of the GBs didn’t change much during the ablation, and some PA12 and GBs still maintained strong interfacial adhesion.

**Figure 10:**
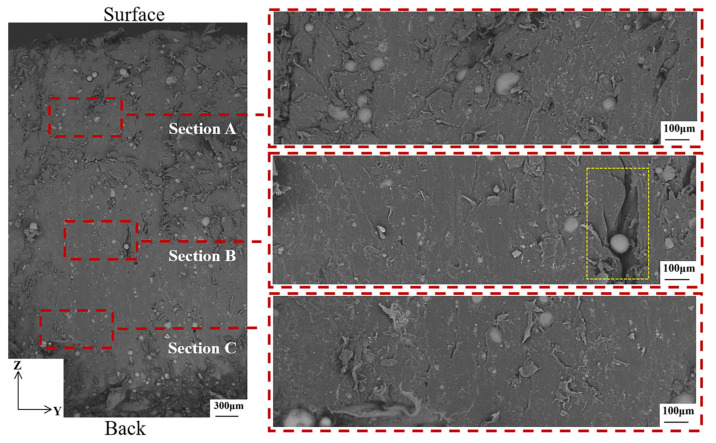
Microscopic morphology of the motor before ablation in the ZOY direction.

Before ablation, the morphology of the GBs and PA12 in the YOZ direction and the XOY direction is similar in Figure 10. For the section marked in section B, the surface of the beads is very clean with no trace of any coating suggesting very poor particle matrix adhesion. Due to the very poor particle matrix adhesion, the load is not transferred to the particles so they do not contribute to Young’s modulus in the expected way.

**Figure 11:**
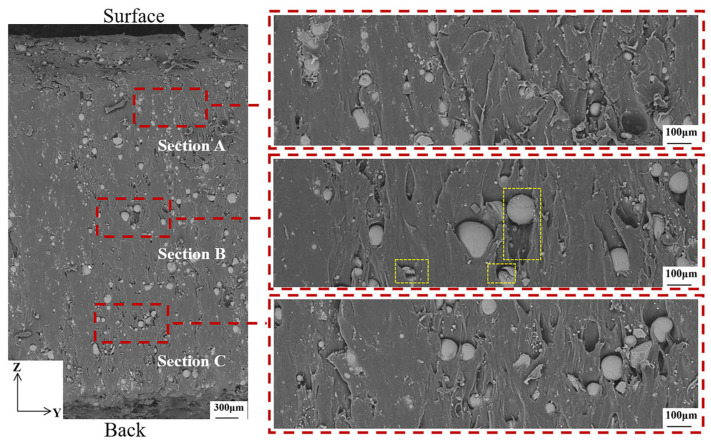
Microscopic morphology of the motor after ablation in the ZOY direction.

After ablation, the morphology of the GBs and the polymer matrix in the ZOY direction is shown in Figure 11. Some of the GBs have shifted or broken as can be seen in section B boxed out. Previous authors have emphasized the importance of strong bond strength between the polymer and the glass in order to achieve the best mechanical properties [14].

In short, the GB with strong interfacial adhesion to PA12 is not affected under the motor ablation process because this part of the material has strong rigidity and is not easily deformed; while the GB with weak or no interfacial adhesion to PA12 may cause fragmentation or displacement, which will make the mechanical properties of the material degrade.

**Figure 12:**
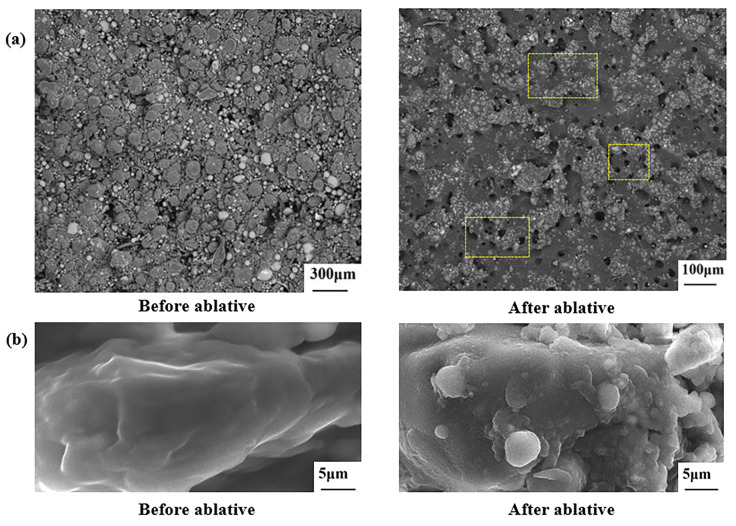
Microscopic appearance. (**a**): the inner wall surface of the motor before and after ablation, (**b**): PA12 before and after ablation.

For a comprehensive investigation and analysis of the motor ablation, the microscopic morphology of the internal wall surface of the motor before and after the ablation was observed. In Figure 12a, the presence of GBs in the composite leads to surface roughness and local protrusion, and there are also a large number of holes in the surface of the composite. Figure 12b shows the surface microscopic morphology of the PA12 before and after ablation. Before ablation, the surface of PA12 was smooth and clean with no obvious deposits. After ablation, the surface of PA12 was very rough. Some surfaces are covered by unstructured spherical deposits and others are encased in lumps.

### 3.3. Analysis of Ablation Process

**Figure 13:**
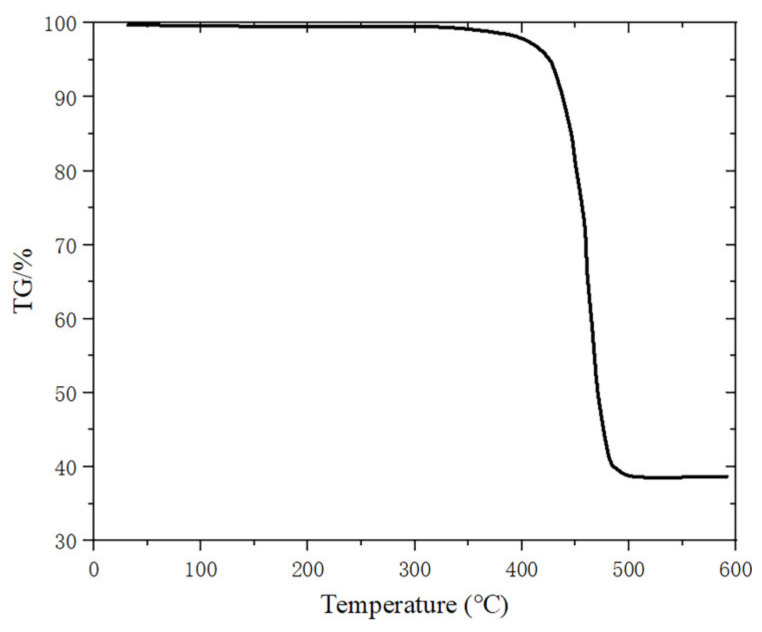
The weight loss rates of PA12/GBs of the outer wall surface.

**Figure 14:**
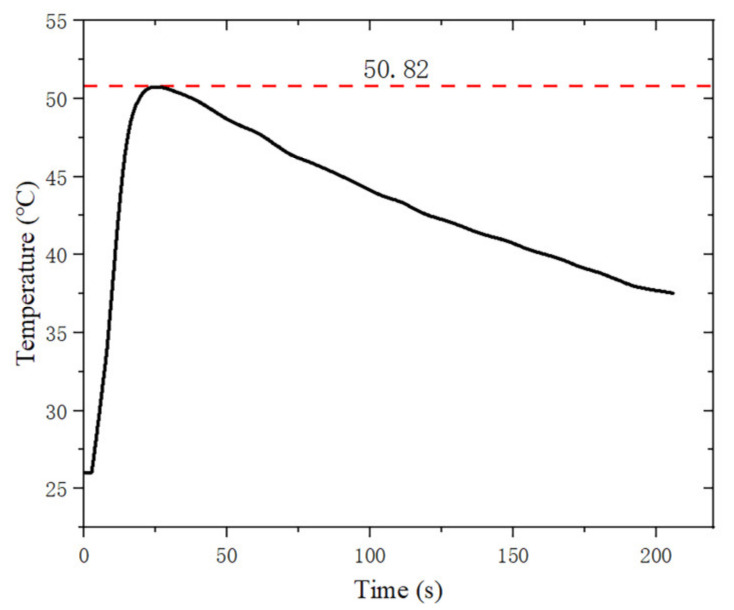
Temperature-time variation curve materials.

The weight loss rates of PA12/GBs materials at 20–600 °C are given in Figure 13. PA12/GBs composite powder exhibited one apparent weight-loss step at 390–500 °C. The temperature of the thermal decomposition peak of the material was about 460 °C. The weight loss rate was 38.4 wt.% after complete thermal degradation. The mass of the motor before ablation was measured to be 8.115 g and its mass after ablation was reduced by 1.411 g. Figure 14 illustrates that during the ablation process, the motor closer to the outer wall surface does not reach the thermal decomposition temperature.

In this paper, the equilibrium composition of propellant combustion products is calculated using the chemical equilibrium products based on the minimum free energy principle. The estimated chamber pressure (12 MPa) is used to calculate the chemical equilibrium state. The results show that the main products of combustion in the combustion chamber are gaseous water, carbon monoxide, hydrogen, carbon dioxide, and nitrogen as shown in Table 4.

EDS analysis was performed on the motors before and after ablation. The C content and O content can’t be used for analysis because the currently limited measurement means lead to no guarantee that the same area is used for EDS analysis before and after ablation. PA12/GBs composite powder has a very low N content and the SG-2 propellant has a high N content. Thus, the analysis was mainly done by N content. In particular, the cross-section of the acquired sample of the engine in the XOY direction is called the transverse section and the cross-section of the acquired sample of the engine in the YOZ direction is called the longitudinal section.

The results of the EDS analysis of the transverse sections in sections A, B, and C are shown in Table 5. The significant increase in the N content of the ablated motors indicated that the thermal decomposition products of PA12/GBs underwent complex chemical reactions with the combustion products of the propellant. The combustion products of the propellant entered the interior of the motor through the pores and underwent complex chemical reactions with the thermal decomposition products of PA12/GBs, but didn’t reach the A region.

The results of EDS analysis of the longitudinal sections of A, B, and C are shown in Table 6. For the B and C regions, the change in content allows us to assume that the thermal decomposition products of PA12/GBs have undergone complex chemical reactions with the combustion products of propellants.

**Figure 15:**
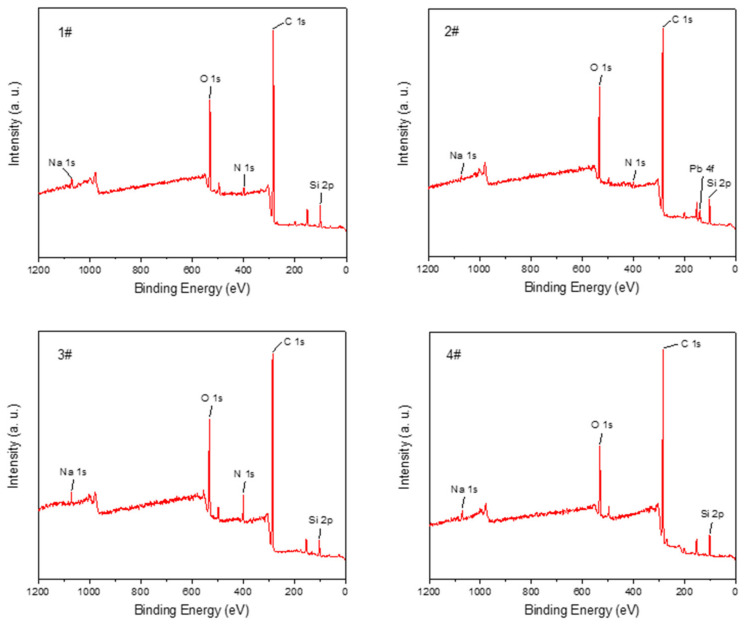
The XPS survey scans of different surfaces. The section of the motor before the ignition 360 test: (**1#**) the longitudinal section, (**2#**) inner wall section, (**3#**) transverse section. (**4#**) is the inner wall section of the motor before the ignition test.

The XPS survey scans of four specimens were performed, as shown in Figure 15. The code number 1#, 2#, and 3# represents the longitudinal section, inner wall section, and transverse section of the motor after the ignition test, respectively. Code number 4# represents the inner wall section of the motor before the ignition test. The samples scanned by XPS are consistent with those scanned by EDS. The scanned area is 0.7 mm × 0.3 mm. The longitudinal and transverse sections are scanned in the middle of their sample surfaces. As can be seen from Figure 15, the peak positions and intensities of most of the elements on the four surfaces match well, but only the inner wall surface has Pb.

The content of each element was obtained by quantitative calculation of the peak areas of the characteristic peaks of the elements in the broad spectrum as shown in Table 7. It can be found that the C element content of the material after the ignition experiment has a decreasing trend, and the O content has an increasing trend. There is basically no N element in the material before the ignition experiment, but there is obvious N element on the surfaces of the three different directions of the engine after the ignition experiment. At the same time, the presence of Pb can be detected on the inner wall surface of the engine after the ignition experiment. PA12/GBs have very little N content, but the propellant has a higher N content. It can be concluded that a complex chemical reaction occurred between the propellant and the composite by combining with the EDS analysis.

**Figure 16:**
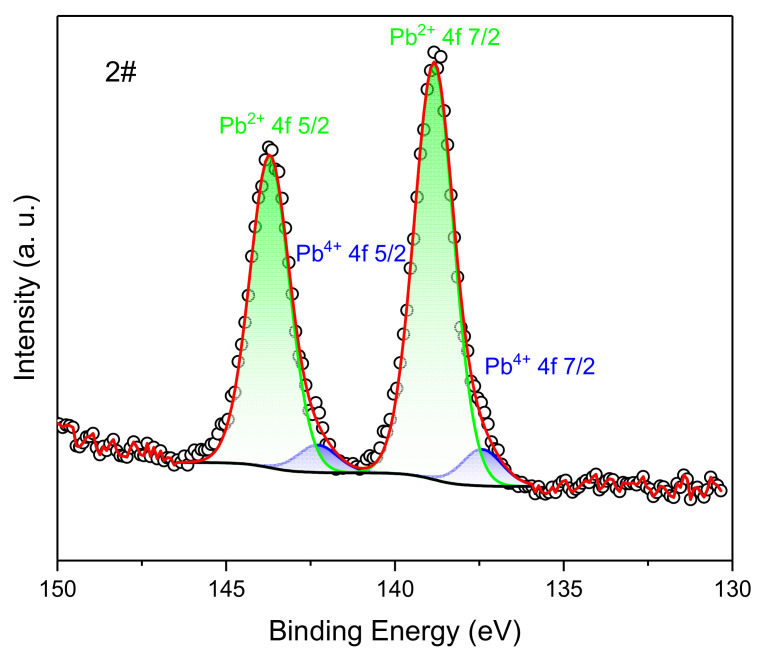
High-resolution XPS spectra in the Pb 4f region of the inner wall surface of the engine after the ignition experiment. The black line is the baseline, the black circle line is the peak of the original data, and the red line is the summation peak of the two valence states after the partial peak fitting. The blue and green areas are the proportions of the different valence states of Pb 4f.

Because only the broad spectrum of the inner wall surface of the engine after the ignition experiment has the characteristic peak of Pb, its fine spectrum scan can obtain information on chemical state composition. The high-resolution XPS spectra of the Pb 4f region for graphite are shown in Figure 16. A split-peak fit to the Pb 4f fine spectrum was performed using CasaXPS 2.3.25PR1.0 software, and the Pb 4f spectrum was fitted to 2 sets of peaks using the Gauss–Lorentz fit method. They were Pb^4+^ 4f _5/2_, Pb^4+^ 4f _7/2_, Pb^2+^ 4f _5/2_, Pb^2+^ 4f _7/2_ in order, and the corresponding binding energies were around 137.4 eV, 142.3 eV, 138.8 eV, and 143.7 eV. The percentage of Pb^2+^ was 8.11% and the percentage of Pb^4+^ was 91.89%. The compounds containing lead mainly exist in the form of Pb^4+^, with only a small portion of Pb^2+^, and there is no presence of monomeric Pb. From Table 3, it was known that the propellant contains 3% of PbCO3. During the ignition experiment, Pb underwent an oxidation reaction and no reduction reaction. The oxidation products obtained from its oxidation reaction were deposited on the surface of the motor’s combustion chamber.

In brief, the composite material underwent thermal decomposition. Moreover, it underwent a complex chemical reaction with the propellant. Part of the material near the outer wall surface did not reach the thermal decomposition temperature.

## 4. Conclusions

These results give a basis for the ablation mechanism of PA12/GBs SRMs under high temperature and pressure conditions, and provide theoretical and practical references for the application of micropropulsion systems for satellites, as follows:

(1) A new SRM is proposed in this paper. Ablation experiments found that the motor can withstand the pressure of 12 MPa and the temperature of 2500 K.
(2) The maximum ablation rate for the motor’s combustion chamber was 0.22 mm/s, which occurred at the location where the combustion chamber meets the baffle. The closer to the nozzle, the greater its ablation rate.
(3) During the ablation process, the GBs with strong interfacial adhesion to PA12 is not affected because this part of the material has strong rigidity, while the GBs with weak or no interfacial adhesion to PA12 may cause fragmentation or displacement, which will make the mechanical properties of the material degrade.
(4) Part of the material near the outer wall surface did not reach the thermal decomposition temperature, so the outer wall temperature is about 324 K.
(5) The ablated motor had a large number of holes and some deposits on the inner wall surface.
(6) The composite material underwent thermal decomposition. Moreover, it underwent a complex chemical reaction with the propellant.

## Figures and Tables

**Table 1:** Values of material parameters.

Parameter	Shell	Nozzle
Density (g·m^−3^)	1.22	7.93
Tensile strength (MPa)	51	595

**Table 2:** Values of process parameters for printing equipment.

Parameter	Value
Laser power (P), W	30
Laser scan speed (V), mm/h	15
Powder deposition thickness (H), mm	0.1
Laser beam diameter (D), mm	0.15
Preheating temperature, °C	170 °C

**Table 3:** SG-2 propellant’s main formulations.

Ingredient	C_6_H_7_O_2_(ONO_2_)_a_(OH)_3-an_	C_3_H_5_N_3_O_9_	C_7_H_6_N_2_O_4_	PbCO_3_
Content (%)	56	27.5	8.5	3

**Table 4:** The propellant combustion products.

Products	CO	CO_2_	H_2_	H_2_O	N_2_
**Mass fraction** (**%**)	41.6	11.1	13.2	21.4	11.8

**Table 5:** Comparison of the elements in each section of the the transverse sections (%).

Section	State	Compound	
		**Si**	**N**
**A**	Before ablation	2.94	0
	After ablation	2.15	0
**B**	Before ablation	3.14	0
	After ablation	2.12	20.81
**C**	Before ablation	3.78	0
	After ablation	1.64	20.97

**Table 6:** Comparison of the elements in each section the the longitudinal sections (%).

Section	State	Compound	
		Si	N
A	Before ablation	1.36	0
	After ablation	1.58	0.05
B	Before ablation	1.09	0
	After ablation	1.27	9.97
C	Before ablation	1.91	0
	After ablation	1.45	10.52

**Table 7:** Comparison of the elements before and after ablation (%).

Sample	Compound					
	C	O	N	Si	Na	Pb
1#	76.89	14.13	1.10	6.47	0.65	0
2#	74.69	14.83	0.99	7.57	0.45	0.23
3#	75.75	14.81	4.2	4.68	0.57	0
4#	77.45	11.60	0	6.40	0.91	0

## Data Availability

Available on request.
